# Comparative Transcriptome Analysis of *Ampelopsis megalophylla* for Identifying Genes Involved in Flavonoid Biosynthesis and Accumulation during Different Seasons

**DOI:** 10.3390/molecules24071267

**Published:** 2019-04-01

**Authors:** Min Yang, Peina Zhou, Chun Gui, Guozheng Da, Ling Gong, Xiuqiao Zhang

**Affiliations:** College of Pharmacy, Hubei University of Chinese Medicine, Wuhan 430065, China; yangminzj@ntu.edu.cn (M.Y.); zhoupeina@163.com (P.Z.); gc19890703@163.com (C.C.); daziwy@163.com (G.D.)

**Keywords:** *Ampelopsis megalophylla*, flavonoid biosynthesis, flavonoid accumulation, transcriptome

## Abstract

*Ampelopsis megalophylla* is an important species used in Chinese folk medicine. Flavonoids, the most important active components of plants, greatly determine the quality of *A. megalophylla*. However, biosynthesis of flavonoids at the molecular and genetic levels in *A. megalophylla* is not well understood. In this study, we performed chemical analysis and transcriptome analysis of *A. megalophylla* in different seasons (i.e., May, August, and October). Accumulation of flavonoids was higher in May than in the other two months. Genes involved in the flavonoid biosynthesis pathway, such as chalcone synthase, anthocyanidin synthase, flavanone 3-hydroxylase, flavonoid-3′,5′-hydroxylase, caffeoyl-CoA *O*-methyltransferase, dihydroflavonol 4-reductase, 4-coumarate-CoA ligase, phenylalanine ammonia-lyase, cinnamate 4-hydroxylase, flavonoid 3′-monooxygenase, shikimate *O*-hydroxycinnamoyltransferase, and leucoanthocyanidin reductase, were identified based on transcriptome data. Fifty ATP binding cassette (ABC) transporter, nine SNARE, forty-nine GST, and eighty-four glycosyltransferases unigenes related to flavonoid transport and biomodification were also found. Moreover, seventy-eight cytochrome P450s and multiple transcription factors (five MYB, two bHLH, and three WD40 family genes) may be associated with the regulation of the flavonoid biosynthesis process. These results provide insights into the molecular processes of flavonoid biosynthesis in *A. megalophylla* and offer a significant resource for the application of genetic engineering in developing varieties with improved quality.

## 1. Introduction

*Ampelopsis megalophylla* Diels et Gilg, which belongs to the family Vitaceae, is a highly prized folk medicinal herb in the Western parts of Hubei Province in China, where it is native. The dried leaves of *A. megalophylla*, also called “Meicha”, are often consumed as herbal tea and have been used for the treatment of hypertension in clinical settings. Modern pharmacology studies indicate that *A. megalophylla* has broad pharmacological functions, including anti-hepatitis B virus (HBV), antimicrobial, antioxidant, and antitumor properties [1,2,3,4]. These properties have been attributed to the characteristic secondary metabolites of *A. megalophylla*, including flavonoids such as ampelopsin (dihydromyricetin), myricetin, and myricitrin [5,6].

In recent years, a sharp decline has occurred in the natural populations of *A. megalophylla*, owing to intensive harvesting of this valuable medicinal plant. It is important to develop methods to artificially cultivate this species in order to protect wild medicinal plant populations and resolve the shortage of this resource. Thus, there is an urgent need to conduct studies focused on improving the quality of *A. megalophylla* grown under artificial conditions. It is well-known that the quality of traditional Chinese herbs depends on their intrinsic active compounds, and the season is known as a crucial factor that influences the formation and accumulation of secondary metabolites in plants. It has been reported that the bud period of *A. megalophylla* begins in April, the defoliating period occurs in November, and the best harvest time for medicinal purposes is May, when the highest level of flavonoids occurs [7]. Understanding the regulation mechanism of formation and accumulation of flavonoids during different seasons may greatly facilitate the development of *A. megalophylla* with high quality.

Flavonoids are a large group of secondary metabolites and have attracted much attention worldwide due to their potential value in the pharmaceutical and nutraceutical industries [8,9]. Studies on the flavonoid biosynthesis pathway of plants have been performed, and it is generally believed that genes associated with the flavonoid synthesis pathway are mainly divided into two categories [10]: Structural genes and regulatory genes. The regulatory genes category includes the MYB-bHLH-WD40 transcription factors. In addition, glycosyltransferases and cytochrome P450s also contribute to flavonoid regulation in plants [11]. However, genes involved in flavonoid biosynthesis in *A. megalophylla* have not been reported, largely due to non-availability of the reference genome sequence and lack of genomic information for this species. In the present study, to better understand the molecular mechanisms of flavonoid biosynthesis during different seasons in *A. megalophylla*, we determined quantitative differences of flavonoids in *A. megalophylla* samples collected at different times of the year. We also performed constituent and transcriptome analysis to reveal the genes associated with flavonoid biosynthesis and accumulation.

## 2. Results

### 2.1. Determination of Flavonoids in A. megalophylla

**Figure 1:**
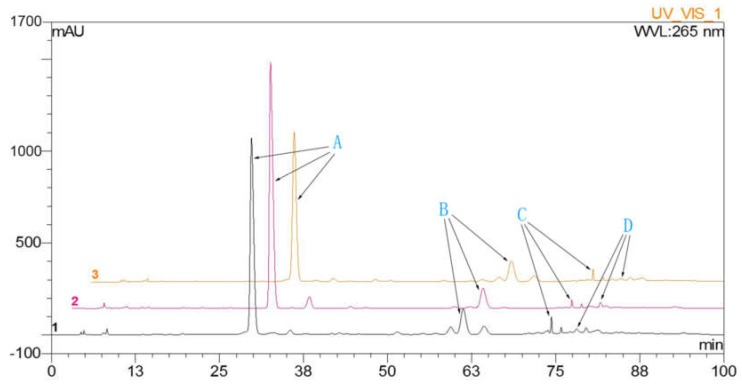
The HPLC chromatographic: A, dihydromyricetin; B, myricitrin; C, quercitrin; D, myricetin.

HPLC analysis was performed to detect dihydromyricetin, myricitrin, quercitrin, and myricetin in leaves of *A. megalophylla* harvested during different seasons (May, August, and October). As shown in Table 1, concentration of these four flavonoids varied between samples collected from the three different months. Dihydromyricetin, myricitrin, and quercitrin contents were higher in May than those in the August and October (Figure 1), whereas myricetin contents were highest in August.

### 2.2. Transcriptome Sequencing and De Novo Assembly

To acquire a comprehensive overview of *A. megalophylla*, cDNA libraries were constructed from leaf samples of *A. megalophylla* harvested in May, August, and October. The libraries were sequenced using Illumina paired-end sequencing technology. After stringent data filtering and quality assessment, approximately 44.2, 58.5, and 47.3 million clean reads were obtained for the transcriptomes in May, August, and October, respectively (Table 2). Then, all the high-quality clean reads were de novo assembled using the Trinity program. A total of 83,886 unigenes, which comprised 68,294,276 bp, were acquired, with an average length of 814 bp, a maximum length of 17,500 bp, and an N50 of 1648 bp (Table S1). The Q30 values and Q20 values were greater than 95.40% and 91.12%, respectively, and GC content ranged from 45.20% to 46.75% (Table 2). The size distribution of these generated unigenes is shown in Figure S1; 74.45% of all unigenes showed lengths ranging from 200 to 1000 bp, unigenes longer than 1000 bp in length accounted for 24.45% of the total, and unigenes shorter than 200 bp accounted for the remaining 1.1%.

### 2.3. Functional Annotation and Classification of A. megalophylla Unigenes

After de novo assembly, 83,886 unigenes were generated. There were differences in the quantity and expression pattern of these unigenes among libraries from samples collected during different months. A total of 28,656 unigenes were expressed in all three months, while 16,379, 10,008, and 11,860 unigenes were expressed specifically in May, August, and October, respectively (Figure S2). The unigenes generated in this study were searched against the following public protein databases: NCBI-NR (National Center for Biotechnology Information-non-redundant protein database), Swiss-Prot, GO (Gene Ontology), Pfam, EggNOG (Evolutionary genealogy of genes: Non-supervised Orthologous Groups), KEGG (Kyoto Encyclopedia of Genes and Genomes), TmHMM (transmembrane protein prediction), and SignalP databases. Among 83,886 unigenes, a total of 50,054 (59.67%) unigene sequences were annotated in the eight public databases. Between them, 41,139 (49.04%), 38,096 (45.41%), 22,788 (27.17%), 19,906 (23.73%), 10,863 (12.95%), 12,639 (15.07%), 8860 (10.56%), and 2967 (3.54%) of the unigenes presented matches in the Swiss-Prot, Blast-NR, GO, Pfam, EggNOG, KEGG, TmHMM, and SignalP databases, respectively (Table 3).

**Figure 2:**
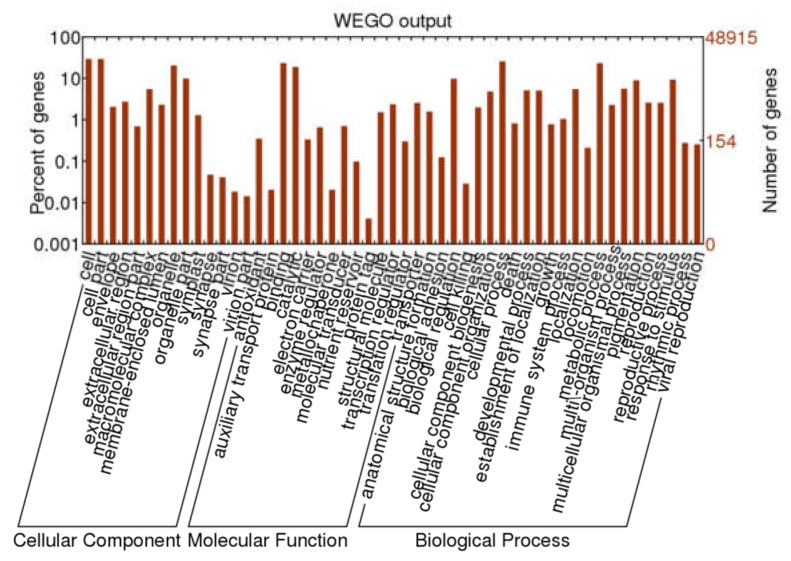
Gene ontology classification of *A. megalophylla* unigenes.

GO annotation was used for classifying the function of unigenes. A total of 22,788 unigenes were grouped into three main functional categories and were then further classified into 52 subgroups, including 14 terms of cellular components (CC), 14 terms of molecular functions (MF), and 24 terms of biological processes (BP). In the CC, a high percentage of unigenes were linked with the terms “cell”, “cell part”, “orangelle”, and “orangelle part”. Most of the GO terms related to MF unigenes were “binding” and “catalytic activity”, followed by “structural molecule” and “transcription regulator”. Most of the assignments in BP were “cell process”, “metabolic process”, “biological regulation”, and “response to stimulus” (Figure 2).

**Figure 3:**
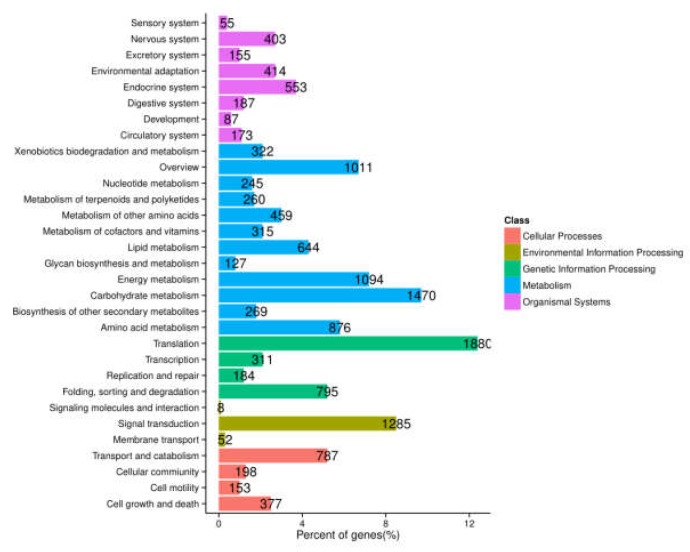
Functional classification and pathway assignment of assembled unigenes by KEGG.

To further determine biochemical pathways, all the unigenes were searched against the KEGG database, and a total of 12,639 entries were annotated. These were classified into five main groups. Among them, the pathways most represented were “metabolism” (7092 unigenes), followed by “genetic information processing” (3170 unigenes), “organismal systems” (2027 unigenes), “cellular processing” (1515 unigenes), and “environmental information processing” (1345 unigenes) (Figure 3). This annotation information could aid further investigation of specific processes, functions, and pathways in *A. megalophylla*.

**Figure 4:**
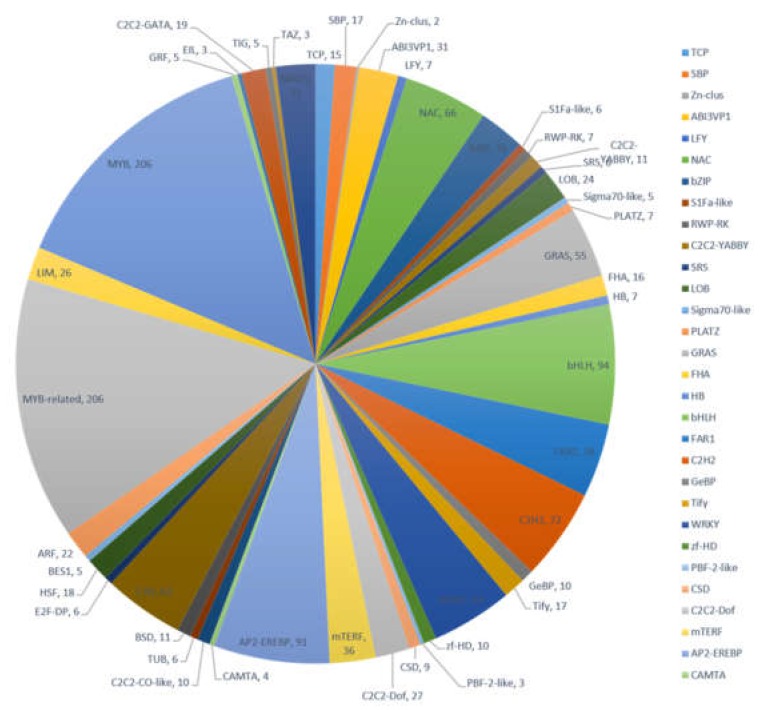
Distribution of transcription factors in *A. megalophylla*.

Transcription factors (TFs) play an important regulatory role in plant functions such as growth, development, and metabolism. Many TF families consist of diverse groups of gene families, including MYB, MYB-related, bHLH, AP2-EREBP, C2H2, and others. Results from our transcriptome data revealed that 1458 transcripts encode potential TFs, which can be sorted into 47 TF families (Figure 4). Members of the MYB and MYB-related transcription factors were the most abundant (206, 14.13%) followed by bHLH (94, 6.45%), AP2-EREBP (91, 6.24%), and C2H2 (72, 4.94%). The identification of TFs provided more abundant information to characterize TFs in various biochemical pathways in *A. megalophylla*.

### 2.4. Differentially Expressed Gene (DEG) Analysis of A. megalophylla

**Figure 5:**
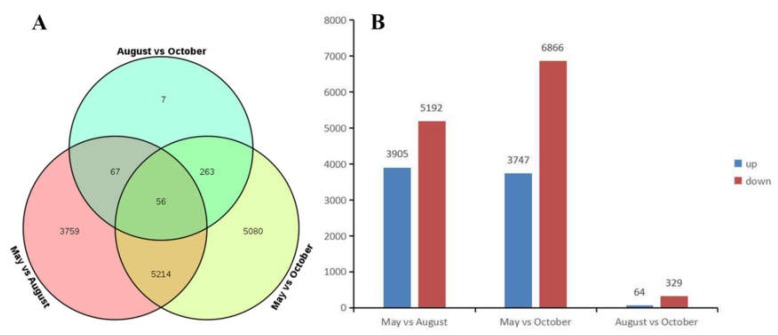
(**A**) The Venn diagram of the number of differentially expressed genes (DEGs); (**B**) The number of up-regulated and down-regulated DEGs.

We explored the transcriptomes of samples to identify differentially expressed genes (DEGs) during different months. Transcripts with > log two-fold differential expression and with false discovery rates (FDRs) of >0.05 were used as a threshold for the DEG analysis. There were 9096 DEGs between the May and August libraries, of which 3905 were up-regulated in May and 5191 were up-regulated in August. There were 10,613 DEGs identified between the May and October libraries, of which 3747 were up-regulated in May and 6866 were up-regulated in October. There were 393 DEGs between the August and October libraries, of which 64 were up-regulated in August and 329 were up-regulated in October (Figure 5).

To elucidate the functions and biological pathways of the DEGs, GO analysis and KEGG pathway analysis were performed. In the GO enrichment analysis, nearly half of the DEGs were assigned to the term “biological process”. Among them, genes linked with the terms “cell process”, “metabolic process”, “response to stimulus”, and “biological regulation” were highly enriched. Within the “cellular component” enriched set, genes linked to the terms “cell”, “cell part”, “organelle”, and “organelle part” were highly enriched. On the other hand, GO terms related to “molecular function”, such as “binding”, “catalytic”, “structural molecule”, and “transcription regulator” were highly enriched depending on the month (Figure S3).

In the KEGG analysis, among 9097 DEGs of the ‘May vs. August’ comparison, 4092 unigenes were mapped into 303 KEGG pathways. There were 301 pathways in the ‘May vs. October’ comparison and 85 pathways in the ‘August vs. October’ comparison. Among these, pathways involved in the cell cycle, various metabolic pathways, and secondary metabolite biosynthesis, such as phenylpropanoids, flavone, and flavonol flavonoid biosynthesis, were significantly enriched.

### 2.5. Candidate Genes Related to Flavonoid Biosynthesis

**Figure 6:**
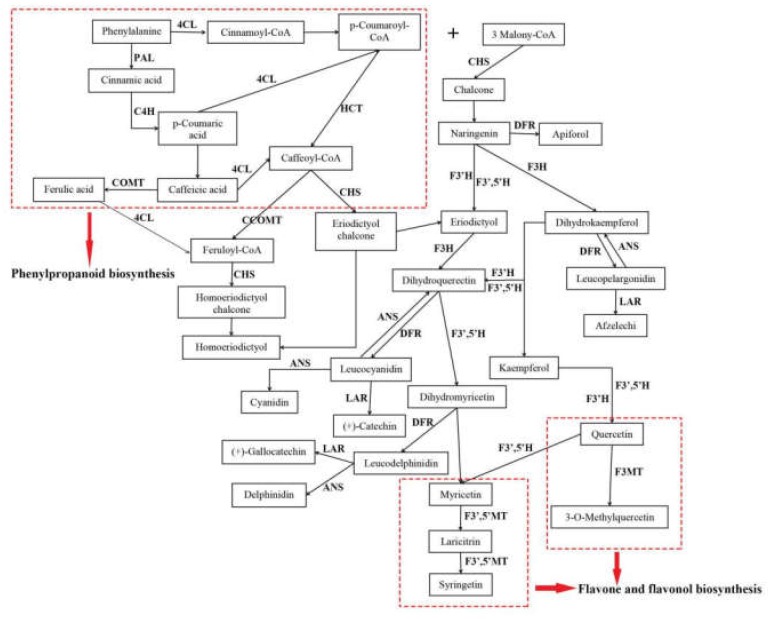
The flavonoid biosynthesis in *A. megalophylla*.

Flavonoids were the main active compounds found in *A. megalophylla* and were important for evaluating its quality. To understand the regulation mechanisms of flavonoid biosynthesis in *A. megalophylla*, key regulatory genes of flavonoid biosynthesis involved in the pathways for phenylpropanoid biosynthesis, flavonoid biosynthesis, and flavone and flavonol biosynthesis were identified in this study. Furthermore, their expression profiles were comprehensively analyzed to assess seasonal variation in expression. The assumed flavonoid synthesis pathway is shown in Figure 6.

**Figure 7:**
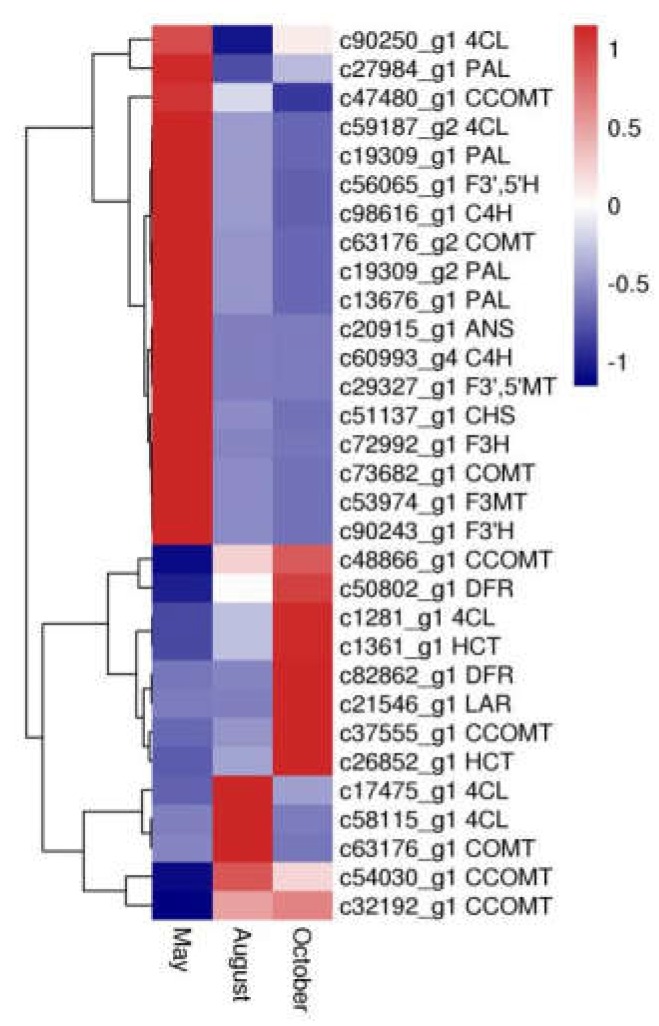
The expression patterns of DEGs involved in flavonoid biosynthesis. Blue and red colors represent different levels of gene expression and the color scales reflect a log_2_-transformed mean of fragments per kilobase of transcript per million mapped (FPKM) values.

Thirty-one unigenes encoding 15 key enzymes observed in this study were mostly related to biosynthesis of flavonoids, including anthocyanidin synthase (*ANS*, EC: 1.14.20.4), flavanone 3-hydroxylase (*F3H*, EC: 1.14.11.9), flavonoid-3′,5′-hydroxylase (*F3′*,*5′H*, EC: 1.14.14.81), caffeoyl-CoA *O*-methyltransferase (*CCOMT*, EC: 2.1.1.104), dihydroflavonol 4-reductase (*DFR*, EC: 1.1.1.219), 4-coumarate-CoA ligase (*4CL*, EC: 6.2.1.12), phenylalanine ammonia-lyase (*PAL*, EC: 4.3.1.24), flavonoid 3-methyltransferase (*F3MT*, EC: 2.1.1.76), cinnamate 4-hydroxylase (*C4H*, EC: 1.14.14.91), flavonoid 3′-monooxygenase (*F3′H*, EC: 1.14.14.82), chalcone synthase (*CHS*, EC: 2.3.1.74), shikimate *O*-hydroxycinnamoyltransferase (*HCT*, EC: 2.3.1.133), leucoanthocyanidin reductase (*LAR*, EC: 1.17.1.3), caffeic acid 3-*O*-methyltransferase (*COMT*, EC: 2.1.1.68), and flavonoid 3′,5′-methyltransferase (*F3′*,*5′MT*, EC: 2.1.1.267) (Table S2). Furthermore, expression pattern analysis showed that these genes were expressed quite differently during different months (Figure 7). In particular, 18 DEGs, including *CHS*, *PAL*, *F3H*, *F3′*,*5′H*, *C4H*, *F3′H*, *ANS*, *F3′*,*5′MT* and *F3MT*, had the highest expression in May, whereas expression of nine DEGs, including *LAR*, *HCT*, and *DFR*, was highest in October.

### 2.6. Candidate Genes Related to Flavonoid Accumulation and Biomodification

The diversity of flavonoids and their distribution in plants depend not only on the expression patterns of key genes in the flavonoid biosynthesis pathway, but also on biomodification, transportation, and accumulation processes. The ATP binding cassette (ABC) transporter, soluble *N*-ethylmaleimide-sensitive factor attachment protein receptors (SNARE), and glutathione S-transferase (GST) have characteristic roles in flavonoid transportation and accumulation [10].

**Figure 8:**
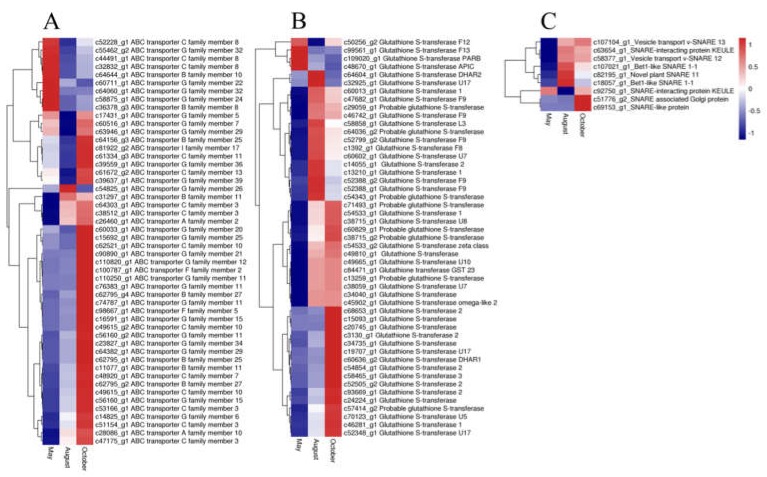
The expression level of DEGs of ATP binding cassette (ABC) transporter (**A**), glutathione S-transferase (GST) (**B**), and soluble *N*-ethylmaleimide-sensitive factor attachment protein receptor (SNARE) (**C**) genes. Blue and red colors represent different levels of gene expression and the color scales reflect a log_2_-transformed mean of FPKM values.

**Figure 9:**
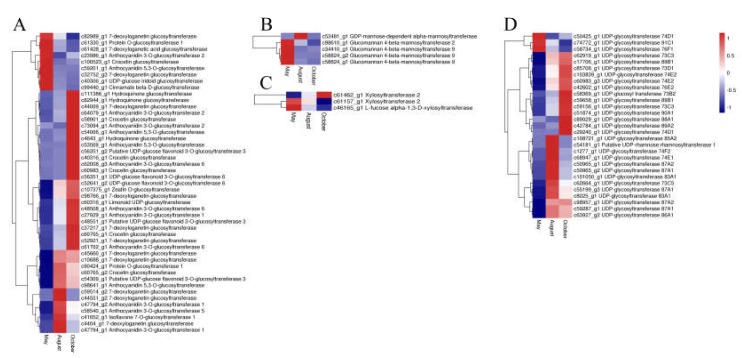
The expression level of glycosyltransferase DEGs including glucosyltransferases (**A**), mannosyltransferases (**B**), xylosyltransferases (**C**), UDP-glycosyltransferases (**D**). Blue and red colors represent different levels of gene expression and the color scales reflect a log_2_-transformed mean of FPKM values.

In our study, 50 ABC transporter unigenes, 9 SNARE unigenes, and 49 GST unigenes were detected. The expression of most of the ABC transporter unigenes was highest in October and lowest in August. Most unigenes of both SNARE and GST had the highest expression in August and October, whereas only a few unigenes had the highest expression in May (Figure 8). Glycosyltransferases (GTs) may also have a vital function for flavonoid accumulation and biomodification. Analysis of the annotation data revealed 84 unigenes for glycosyltransferases. Among these genes, 29, 47, 5, and 3 unigenes were annotated to UDP-glycosyltransferases (UGTs), glucosyltransferases, mannosyltransferases and xylosyltransferases, respectively. The expression patterns of the GTs are shown in Figure 9. The glucosyltransferases and UDP-glycosyltransferases had the same expression levels. Most of the genes were highly expressed in August or October, whereas genes encoding mannosyltransferases and xylosyltransferases were highly expressed in May.

### 2.7. Expression of Transcription Factors and P450 Family Genes Related Flavonoid Biosynthesis and Transport

**Figure 10:**
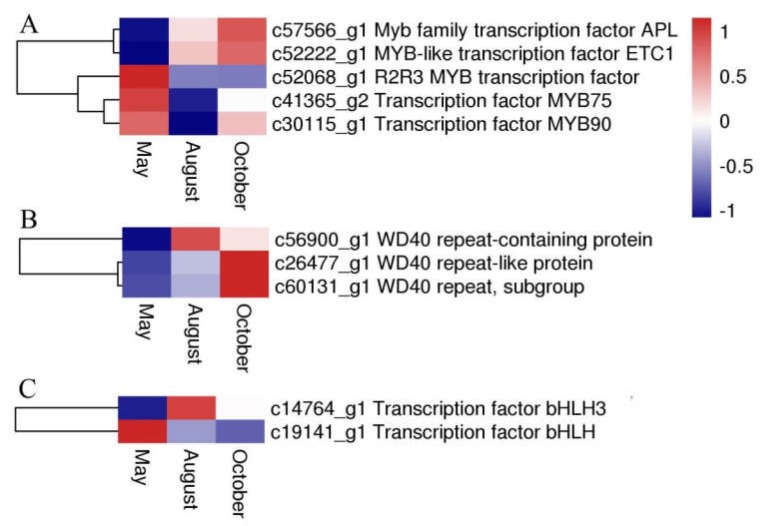
The expression level of DEGs encoding MYB (**A**), WD40 (**B**), and bHLH (**C**). Blue and red colors represent different levels of gene expression, and the color scales reflect a log_2_-transformed mean of FPKM values.

Several flavonoid biosynthesis and transport genes are known to be transcriptionally regulated by TFs belonging to the MYB, bHLH (basic helix-loop-helix), and WD40 families [12,13]. These proteins usually act as a MYB-bHLH-WD40 (MBW) complex to regulate the biosynthetic pathway of flavonoids. In our analysis, five DEGs were annotated to MYB, two DEGs were annotated to bHLH, and three DEGs encoded WD40 repeat proteins. The expression levels or patterns of these three proteins are shown in Figure 10.

**Figure 11:**
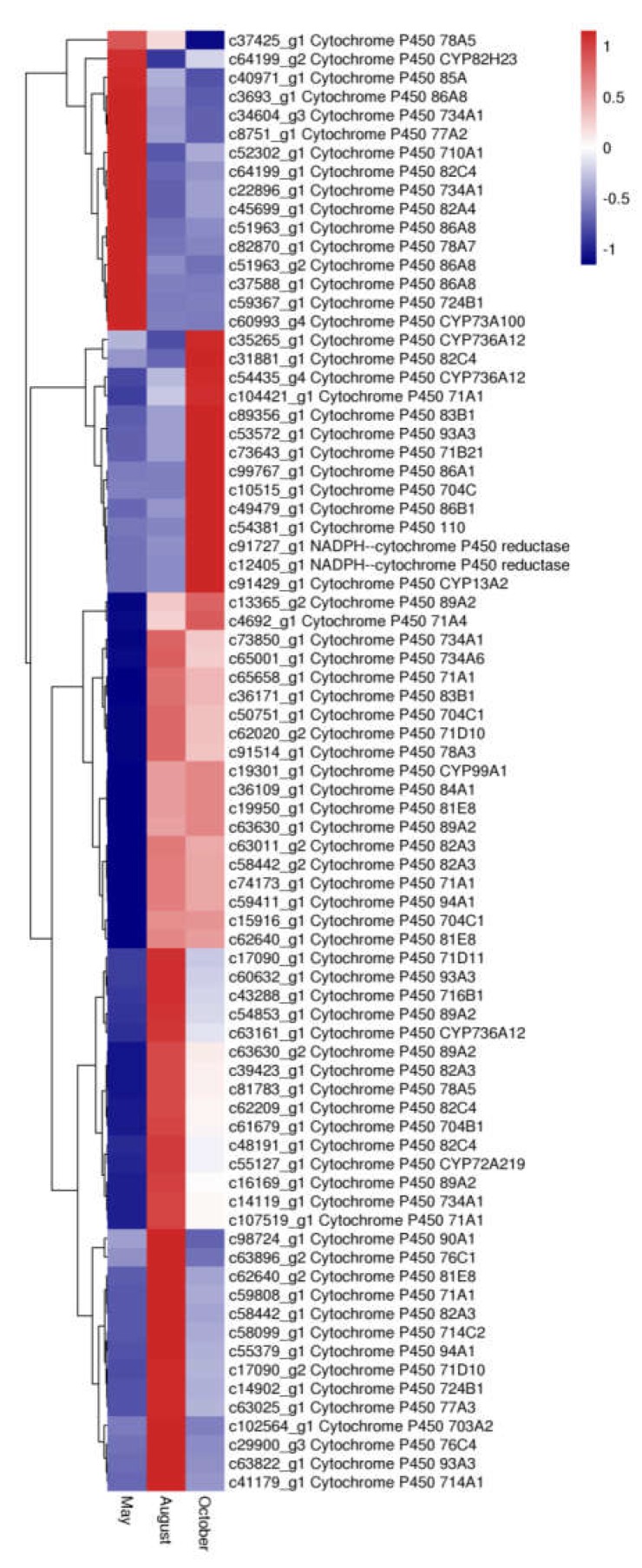
The expression level of DEGs of Cytochromes P450 in *A. megalophylla*. Blue and red colors represent different levels of gene expression, and the color scales reflect a log_2_-transformed mean of FPKM values.

Cytochrome P450s (CYP450s), which comprise one of the largest and oldest gene superfamilies, are known to be involved in the biosynthesis of secondary metabolites such as flavonoids. In this study, 78 unigene DEGs were annotated as CYP450s. Most of these genes belonged to CYP71 (10 genes), nine of them belonged to CYP82, seven of them belonged to CYP704, six of them belonged to CYP86, and four of them belonged to CYP89. The expression levels of the P450 genes among the three months are shown in Figure 11. Sixteen P450 genes were most highly expressed in May, 42 P450 genes were most highly expressed in August, and twenty P450 genes were most highly expressed in October.

### 2.8. Validation of DEGs by qPCR

**Figure 12:**
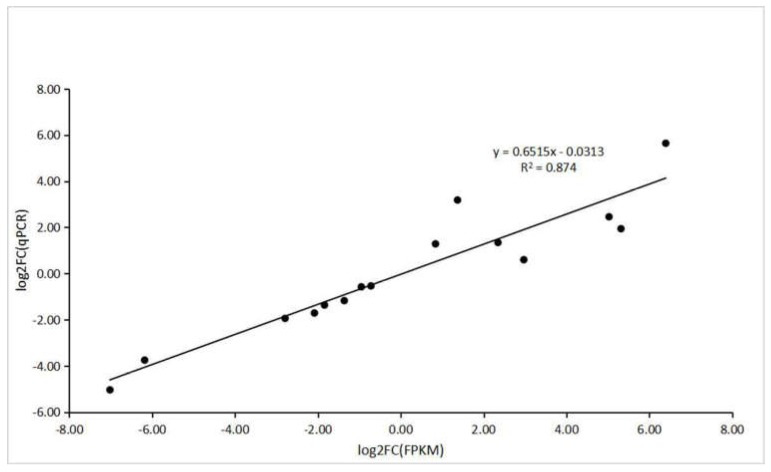
The correlation analysis between RNA-seq and qRT–PCR.

To verify the accuracy of RNA-seq and computational analysis, five DEGs were selected randomly for qRT–PCR. The specific primers used for this analysis are shown in Table S3. All the genes selected for qRT–PCR analysis and those derived from sequencing had a similar trend in expression levels. In addition, correlation analysis between RNA-Seq and transcriptional data of these genes showed a positive correlation (R^2^ = 0.874) (Figure 12).

## 3. Discussion

The plant genus *Ampelopsis* contains about 30 species in the world and 17 species in China [14]. Most of them have medicinal value and biological activity because of their many active ingredients [14]. Within the *Ampelopsis* genus, *A. megalophylla* has been used as folk medicine for hundreds of years in China. Flavonoids are the most important active constituents in *A. megalophylla*. Flavonoids determine the quality of the plants, and flavonoid content varies across the season. However, the molecular mechanism of flavonoid biosynthesis and accumulation that causes seasonal variation in *A. megalophylla* flavonoid content remains largely unexplored. In the present study, we collected *A. megalophylla* samples during three different months and detected quantitative differences in flavonoids between the samples. The results showed that flavonoid contents in samples harvested in different months were vastly different from one another. The characterized flavonoids, including dihydromyricetin, myricitrin, and quercitrin, were found in greatest concentrations in May, followed by August, and were found in the lowest concentrations in October. To explore the reasons for these differences, RNA-Seq technology was used. In transcriptional analysis, a large number of transcripts exhibited a season-specific response. The number of DEGs in the ‘May vs. October’ and ‘May vs. August’ comparisons was much higher than that in the ‘May vs. October’ comparison. These results may imply that biological functions of *A. megalophylla* were extremely active in May.

Various key genes involved in regulation of flavonoid biosynthesis in higher plants have been reported [15,16]. The phenylpropanoid biosynthesis pathway (PATH: ec00940, KEGG) is the foundation to the synthesis of flavonoids, flavones, and flavonols. Cinnamoyl-CoA and 4-cinnamoyl-CoA Coumarin are the precursors of flavonoid synthesis. Their biosynthesis is regulated by related enzymes, such as *PAL*, *C4H*, and *4CL* [17,18], in phenylpropanoid pathways. Subsequently, chalcone is formed from 4-coumaroyl-CoA by *CHS*. This process is recognized as the first rate-limiting step in flavonoid biosynthesis [19]. After this step, different flavonoid subgroups are produced through modifying the molecular backbone, which is controlled by enzymes like *CHS*, *F3H*, *F3′H*, *F3′*,*5′H*, *F3MT*, *F3′*,*5′MT*, *LAR*, *DFR*, *ANS*, *COMT*, *CCOMT*, and *HCT*, which belong to phenylpropanoid biosynthesis, flavonoid biosynthesis (PATH: ec00941, KEGG), and flavone and flavonol biosynthesis (PATH: ec00944, KEGG) [16,17]. For example, methylation of flavonoids by *F3MT* and *F3′*,*5′MT*, compared with nonmethylated flavonoids, can increase their metabolic stability, oral bioavailability, and biological activities [17]. In this work, homologous unigenes of the above genes were identified (Table S2). We also investigated the expression levels of these genes. Interestingly, DEGs encoding *PAL*, *C4H*, *CHS*, *F3H*, *F3′*,*5′H*, *F3′H*, *ANS*, *F3′*,*5′MT*, *F3MT* were more highly expressed in the samples from May than those from the other two months, and the highest flavonoid contents were also in the samples from May. This suggests that flavonoid biosynthesis in *A. megalophylla* may be significantly correlated with expression of these enzymes. It is noteworthy that in addition to high expression of *CHS*, expression levels of *F3′*,*5′H*, *F3H* were also found to be high in May. It has been previously reported that *F3H* catalyzes eriodictyol into dihydroquercetin, which is the precursor of dihydromyricetin [20], and *F3′*,*5′H* converts dihydroquercetin into dihydromyricetin [21], which is a main constituent in *A. megalophylla.* Therefore, *F3H* and *F3′*,*5′H* may be specific genes for dihydromyricetin synthesis in *A. megalophylla.*

The biosynthesis process in plants is sophisticated. Apart from the flavonoid biosynthesis pathway, there are also some genes involved in the biomodification, transportation, and accumulation processes of flavonoids. These genes include SNARE, ABC transporter, GST, GTs, CYP450, and the MYB-bHLH-WD40 complex.

ABC transporters are considered to be involved in the secretion of (iso)flavonoids from soybean roots and may regulate isoflavonoid levels in legumes [22]. SNARE proteins generate energy required for membrane fusion and participate in vesicle-mediated secretion during exocytosis and endocytosis [23]. GST protein, an important flavonoid transporter, is believed to be associated with transportation and separation of anthocyanins in vacuoles [24,25]. These genes were found in the present study and showed different expression patterns. Further study is warranted on the relationship between the effects of ABC transporter, SNARE, and GST gene expression on flavonoid content.

GTs are responsible for the glycosylation process, an important part of the flavonoid modification process, which is essential for producing tremendous chemical diversity and stable accumulation of flavonoids [26,27,28,29]. The ectopic expression of an *Arabidopsis* glycosyltransferase gene UGT76E11 resulted in increased flavonoid accumulation and tolerance to abiotic stresses in transgenic plants [30]. In addition, the biosynthesis and chemical diversity of flavonoid glycosides partly depended on UGTs [31,32]. Among the 29 UGTs in the present study, 26 unigenes were up-regulated in August and October and 3 unigenes were up-regulated in May. Because flavonoid content was highest in May, this suggests that the latter three unigenes were related to flavonoid accumulation in *A. megalophylla*. UGTs are consistently found in plants and particular roles of the DEGs require further investigation.

Cytochrome P450s are known to play central roles in the evolution of metabolic complexity [33] and in the production of secondary metabolites, including flavonoid metabolites [34]. In *Oroxylum indicum*, 31 diverse full length CYP450 genes participate in the biosynthesis of baicalin, baicalein, and other special metabolites [17]. Two CYP450s (CfCYP93B and CfCYP706C) isolated from *Coleus forskohlii* are involved in the biosynthesis of flavonoids [35] and CYP93B isolated from hybrid *Gerbera* encodes flavone synthase II, which catalyzes flavanones into flavones [36]. The expression of CYP450s also has a significant effect on the content of flavonoids in plants [37]. In addition, they, together with other enzymes like *CHS*, *CHI*, drive flavonoid metabolism and plant-specific diversity in a flavonoid metabolon organization [38]. However, further research is needed to understand the exact role of CYP genes involved in the biosynthesis of certain flavonoids. Finally, the ternary MYB-bHLH-WD40 (5 MYB, 2 bHLH, 3 WD40) complex responsible for regulating flavonoid metabolic pathways is well characterized and is highly conserved throughout the plant kingdom. This complex activates and regulates the downstream gene expression of flavonoid metabolism, promotes the biosynthesis of procyanidins, and regulates the biosynthesis of procyanidins at the transcriptional level [39,40]. Therefore, the MBW complex in *A. megalophylla*, like in other plant species, may also play important roles in controlling the biosynthesis and accumulation of flavonoids.

The findings of this study may provide valuable genetic resources for further studies on species diversity of *A. megalophylla* and lay the foundation for studying marker genes to select cultivation with high contents of flavonoids and improve plants on the basis of genome level using genetic engineering. Future studies should explore the specific roles of the genes involved in flavonoid biosynthesis and accumulation in plants

## 4. Materials and Methods

### 4.1. Plant Materials

*A. megalophylla* were collected from Laifeng County, Enshi Tujia and Miao Autonomous Prefecture, Hubei Province, China (29°36.32′N, 109°15.98′E) on 10th May, 21th August, and 14th October. They were three materials of leaves from the same plant. The plant materials were identified by Prof. Xiuqiao Zhang (Hubei University of Chinese Medicine). The leaves of *A. megalophylla* were immediately frozen in liquid nitrogen and stored at −80 °C prior to processing.

### 4.2. Extraction and Determination of Flavonoids in A. megalophylla

The samples collected in May, August, and October were dried and ground into powder. Powder samples (0.5 g) were treated for 30 min by ultrasonic extraction after soaking in petroleum ether (150 mL) for 30 min. The homogenates were filtered, and residues were immersed in 70% alcohol (20 mL) for 30 min and extracted with 70% alcohol by ultrasonic treatment for 30 min. For each extract, 2 mL was dissolved in 3.0 mL of methanol and filtered through a 0.45 µm microporous film. Then, 20 µL of filtrate was run on a Dionex-P680 HPLC (Dionex, Pliening, Germany) system with an Agilent TC-C18 column (250 mm × 4.6 mm, 5 µm, Agilent Technologies Inc., Palo Alto, AR, USA). The mobile phase consisted of acetonitrile (A solvent) and 0.1% phosphate solution (B solvent), used according to the following gradient elution program: 0 min, 0%A/90%B; 15 min, 15%A/85%B; 45 min, 15%A/85%B, 10 min, 25%A/75%B; 15 min, 25%A/75%B and 15 min, 10%A/90%B. The detection wavelength was set at 280 nm, and flow rate was 1 mL/min. The chemical standards included dihydromyricetin (15092141), myricitrin (11060222), and myricetin (14101531), which were purchased from Shanghai Yuanye Biotechnology Co., LTD, Shanghai, China, and quercetrin (20140318), which was purchased from Shanghai Jinsui Biotechnology Co., LTD.

### 4.3. RNA Sequencing and De Novo Assembly

Total RNAs were extracted using RNAprep Pure Plant Kit (Tiangen, Beijing, China) following the manufacturer’s instructions. The RNA concentration and quantity were analyzed using a Nanodrop 2000 (Thermo Fisher Scientific, Waltham, MA, USA) and an Agilent 2100 (Agilent Technologies Inc., Palo Alto, AR, USA). A Stranded Total RNA Library Prep Kit (Illumina, Inc., San Diego, AR, USA) was used for cDNA library construction and normalization. The cDNA library was sequenced by Illumina HiSeq 2000 platform (Illumina, Inc., San Diego, AR, USA). Raw reads were filtered to obtain clean reads by removing sequences with ambiguous base pair identifications (‘N’) and removing low-quality sequences. Then, high-quality clean reads were assembled into unigenes using Trinity software (v.2.4.0, the Broad Institute, Cambridge, MA, USA).

### 4.4. Functional Annotation and Sequence Analysis

The functions of unigenes were annotated using BLAST alignment of the sequences against the Swiss-Prot protein database, NCBI-NR, KEGG, and EggNOG. GO annotation was made by Blast2GO program (v.2.5, BioBam Bioinformatics, Valencia, Spain). These sequences were further analyzed with TMHMM (transmembrane protein prediction) to predict their protein transmembrane regions, and with SignalP to predict signal peptides. The transcription factor (TF) families were identified using known plant transcription factors identified in the PlnTFDB database based on the annotation.

### 4.5. Analysis of Unigene Differential Expression Genes (DEGs)

The FPKM (fragments per kilobase of transcript per million mapped) method was used to normalize and calculate gene expression. The DEGs were screened using the DESeq R package with the threshold false discovery rate (FDR) of < 0.05 and the absolute value of log_2_FoldChange > 1. Singular enrichment analysis of DEGs was carried out to identify the enriched Gene Ontology terms in all comparative conditions with DEGs at a significance level of 0.05. Then, function analysis of the DEGs was also performed using the KEGG pathway.

### 4.6. Quantitative Real-Time PCR

The transcription levels of five putative key enzyme genes were investigated by real-time PCR using an iCycler iQ Real-Time PCR Detection System (Bio-Rad Laboratories, Hercules, CA, USA ) and using the Quant One Step RT-PCR kit (SYBR Green) (Tiangen, Beijing, China) with three replicates. Amplification reactions were performed with the following program: 50 °C for 30 min, 95 °C for 2 min, followed by 40 cycles of 94 °C for 20 s, 55 °C for 20 s, and 68 °C for 20 s. The expression level was normalized to the internal control gene GAPDH and calculated as 2^−ΔΔ*C*t^. All amplification primers are provided in Table S3.

### 4.7. Statistical Analysis

The data were expressed as the mean ± SD of the five putative key enzyme genes from *A. megalophylla* plants collected during different months, and the relative expressions were calculated using the 2^−ΔΔ*C*t^ method. Significance tests were evaluated by one-way ANOVA. The results were analyzed statistically for significance (*p* < 0.05) using IBM SPSS Statistics 22.0 software (IBM Corp., Armonk, NY, USA).

## Figures and Tables

**Table 1:** The content of active component in *Ampelopsis megalophylla* (mg/g, $\bar{\chi }$ + D, *n* = 3).

Samples	Dihydromyricetin	Myricitrin	Quercitrin	Myricetin
May	331.226 ± 1.976	20.889 ± 0.518	2.334 ± 0.025	2.455 ± 0.353
August	277.761 ± 4.908	15.836 ± 0.127	1.202 ± 0.0319	2.843 ± 0.482
October	220.605 ± 1.652	18.308 ± 0.328	1.659 ± 0.100	1.786 ± 0.152

**Table 2:** Summary of transcriptomes from leaf in *A. megalophylla*.

Item	Sample	Read Number	Base Number (bp)	GC Content	Q20	Q30
Raw data	S-5	44,211,695	5,526,461,833	45.83%	95.31%	91.25%
	S-8	58,464,607	7,308,075,917	46.19%	95.75%	92.07%
	S-10	47,325,290	5,915,661,250	46.17%	95.10%	90.86%
Clean data	S-5	43,135,219	5,391,902,417	45.88%	95.74%	91.71%
	S-8	57,752,125	7,219,015,583	46.23%	96.13%	92.46%
	S-10	45,535,950	5,691,993,750	46.15%	95.51%	91.32%

**Table 3:** Summary of the annotations for the assembled unigenes in public databases.

Category	Account	Mapped Ratio (%)
Annotated	50,054	59.67%
Swiss-Prot	41,139	49.04%
Blast-NR	38,096	45.41%
GO	22,788	27.17%
Pfam	19,906	23.73%
KEGG	12,693	15.07%
EggNOG	10,863	12.95%
TmHMM	8860	10.56%
SignalP	2967	3.54%
All	83,886	100.00%

## Supplementary Materials

The following are available online. Figure S1: Length distribution of unigenes from samples of *A. megalophylla*; Figure S2: Venn diagram of unigenes involved in the three different time of *A. megalophylla*; Figure S3: Gene ontology classification of DEGs in *A. megalophylla*; Table S1: The specific primers of qRT–PCR; Table S2: Assembly results of clean reads; Table S3: Details of DEGs related to biosynthesis of flavonoid.
