# Supplementary material for: Comparative Transcriptome Analysis of Ampelopsis megalophylla for Identifying Genes Involved in Flavonoid Biosynthesis and Accumulation during Different Seasons
Source: Molecules. 2019 Apr 1;24(7):1267. doi: 10.3390/molecules24071267 (PMC6480179; doi:10.3390/molecules24071267)
Supplement: Supplementary file 1 [file molecules-24-01267-s001.zip › Supplement information-revised/Supplementary figures.docx]

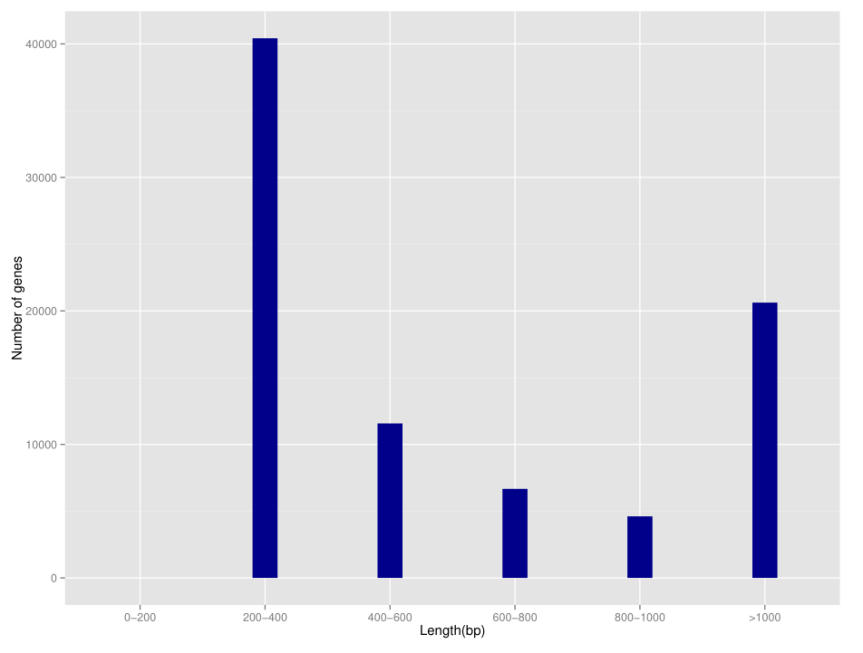


**Figure S1.** Length distribution of unigenes from samples of *A. megalophylla*

*
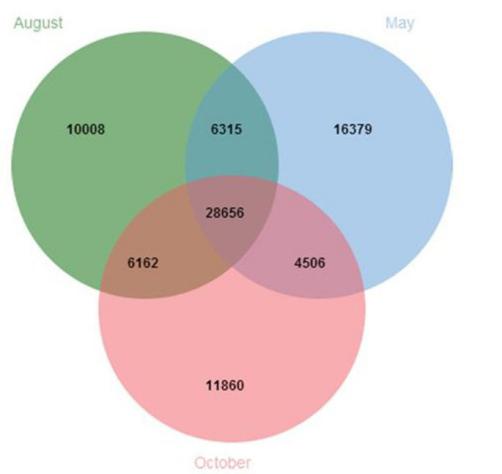
*

**Figure S2.** Venn diagram of unigenes involved in the three different time of *A. megalophylla*


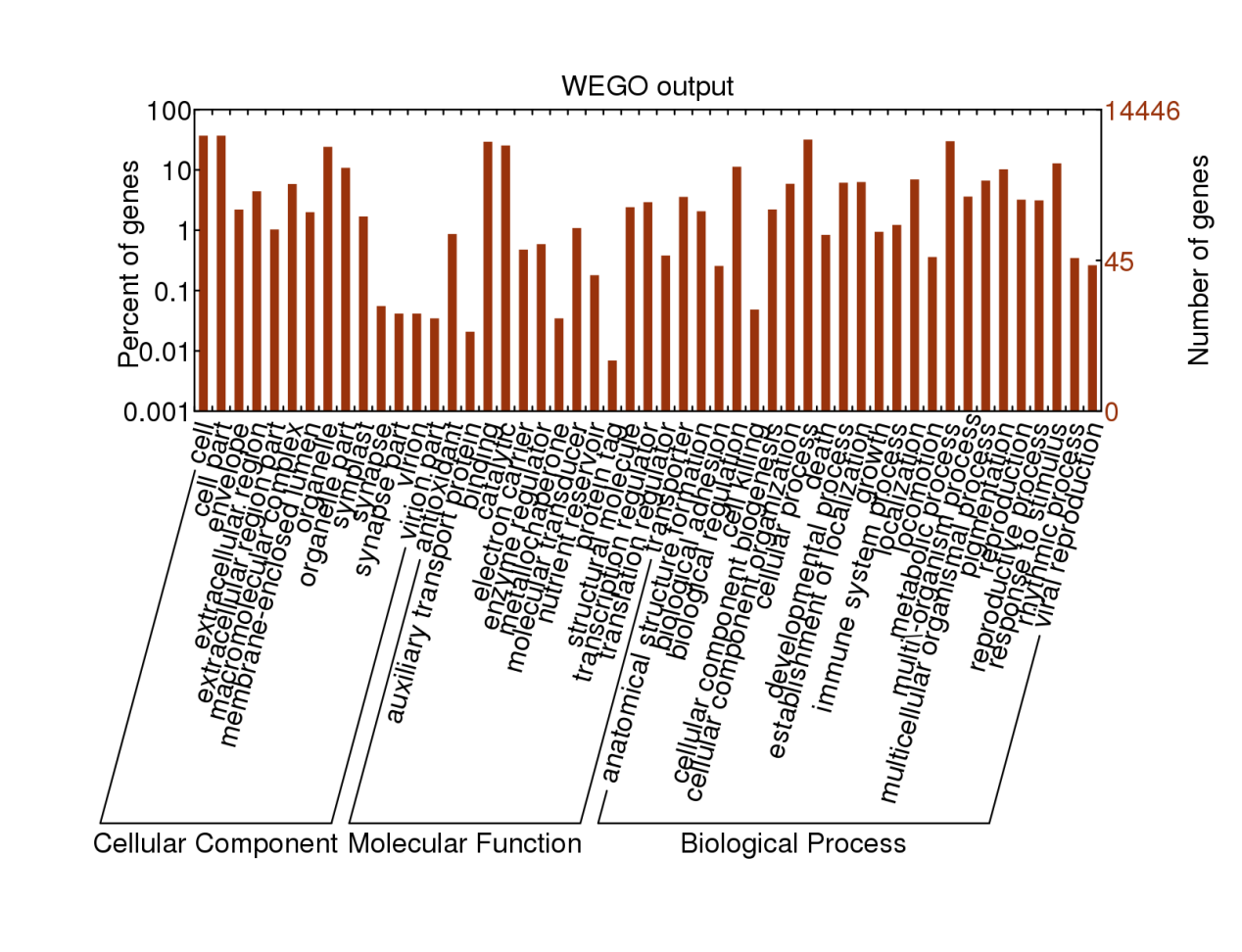


**Figure S3**. Gene ontology classification of DEGs in *A. megalophylla*
